# Polygenic Scores for Height in Admixed Populations

**DOI:** 10.1534/g3.120.401658

**Published:** 2020-09-02

**Authors:** Bárbara D. Bitarello, Iain Mathieson

**Affiliations:** Department of Genetics, Perelman School of Medicine, University of Pennsylvania, Philadelphia, PA, 19104

**Keywords:** admixture, height, polygenic scores, ancestry, Genomic prediction, Shared data resources, GenPred

## Abstract

Polygenic risk scores (PRS) use the results of genome-wide association studies (GWAS) to predict quantitative phenotypes or disease risk at an individual level, and provide a potential route to the use of genetic data in personalized medical care. However, a major barrier to the use of PRS is that the majority of GWAS come from cohorts of European ancestry. The predictive power of PRS constructed from these studies is substantially lower in non-European ancestry cohorts, although the reasons for this are unclear. To address this question, we investigate the performance of PRS for height in cohorts with admixed African and European ancestry, allowing us to evaluate ancestry-related differences in PRS predictive accuracy while controlling for environment and cohort differences. We first show that the predictive accuracy of height PRS increases linearly with European ancestry and is partially explained by European ancestry segments of the admixed genomes. We show that recombination rate, differences in allele frequencies, and differences in marginal effect sizes across ancestries all contribute to the decrease in predictive power, but none of these effects explain the decrease on its own. Finally, we demonstrate that prediction for admixed individuals can be improved by using a linear combination of PRS that includes ancestry-specific effect sizes, although this approach is at present limited by the small size of non-European ancestry discovery cohorts.

Genome-wide association studies (GWAS) have proved remarkably successful at identifying the genomic basis of complex traits. For example, 3,290 genome-wide significant loci explain approximately 25% of the phenotypic variation in height in European ancestry individuals (Yengo *et al.* 2018). This polygenic architecture is a feature of most common diseases (Watanabe *et al.* 2019). One approach to incorporate this information into clinical care is to use polygenic risk scores (PRS). PRS are simply sums of the risk alleles carried by an individual weighted by their effect sizes (Purcell *et al.* 2009). For some diseases (for example, coronary artery disease and breast cancer), PRS can identify individuals with clinically actionable levels of risk (Machiela *et al.* 2011; Mavaddat *et al.* 2015; Torkamani *et al.* 2018; Khera *et al.* 2018).

One major limitation is that the majority of participants in GWAS used to derive PRS are of European ancestry (Popejoy and Fullerton 2016; Sirugo *et al.* 2019). Although many genome-wide significant GWAS hits do replicate in non-European ancestry cohorts (N’Diaye *et al.* 2011; Ng *et al.* 2013; Marigorta and Navarro 2013; Adeyemo *et al.* 2015; Visscher *et al.* 2017), the predictive power of PRS is lower and decreases with genetic distance from Europeans (Márquez-Luna *et al.* 2017; Ware *et al.* 2017; Veturi *et al.* 2019; Martin *et al.* 2019; Duncan *et al.* 2019; Marnetto *et al.* 2020). As a result, the clinical utility of PRS has been explored mainly in European ancestry populations, and little is known about the biological and methodological factors influencing prediction in non-Europeans (Martin *et al.* 2017, 2019; Torkamani *et al.* 2018; Curtis 2018). Such factors may include inter-cohort differences in data collection, phenotype or environment, differences in linkage disequilibrium (LD) structure or allele frequencies across populations, differences in causal or marginal effect sizes, and epistatic or gene-environment interactions (Novembre and Barton 2018).

Simulations have shown that some reduction in predictive power is expected due to differences in allele frequencies and LD patterns across populations (Martin *et al.* 2017; Wang *et al.* 2020). However, there remains a gap between empirical observations, and theoretical and simulation results in that the extent to which these factors explain the observed decrease in real data are unknown.

Here, we address this gap in two ways. First, we describe the reduction in the predictive power of height PRS as a function of ancestry in populations of recently admixed African and European ancestry. Height is a well-studied model for understanding complex polygenic traits, and admixed populations allow us to investigate predictive power as ancestry varies continuously, while controlling for environmental or methodological differences between cohorts (Marnetto *et al.* 2020). Second, we explore the roles of different biological and statistical factors in driving this reduction. Our results suggest that there is no simple statistical solution to the PRS transferability problem and emphasize the importance of performing GWAS in diverse populations.

## Methods

### Data preparation, QC, and phasing

We obtained genotype and phenotype data from the UK Biobank (Bycroft *et al.* 2018) (UKB), the Women’s Health Initiative (Hays *et al.* 2003) (WHI), Jackson Heart Study (Taylor 2005) (JHS), and Health and Retirement Study (Sonnega *et al.* 2014) (HRS) cohorts through dbGaP. For HRS and UKB we also obtained imputed genotype data, described elsewhere (Sonnega *et al.* 2014; Bycroft *et al.* 2018). For WHI and JHS we lifted over SNP positions to hg19 using *liftOver* (https://genome.ucsc.edu/cgi-bin/hgLiftOver). For WHI, JHS, and HRS, we flipped alleles to the positive strand using the appropriate strand files from https://www.well.ox.ac.uk/∼wrayner/strand/. Because the different cohorts each contain different ancestry groups, we initially identified individuals with admixed African ancestry in each cohort using a combination of genetic clustering and self-reported ancestry as described below. For these individuals, we then inferred local ancestry, as described in the next section.

**UKB**: This dataset contains several ancestry groups. We selected 8,813 individuals with African or admixed African and European ancestry-based on PCA **(**Figure S1**)**. After ancestry inference, we further filtered this set to contain individuals with at least 5% genome-wide African ancestry proportion and phenotype availability, resulting in 8,700 individuals that we refer to as UKB_afr (Table 1). We randomly selected 9,998 European ancestry individuals from the “White British” subset to use as a comparison sample and refer to them as “UKB_eur”.

**Table 1:** Datasets used in this study. UKB, UK Biobank; WHI, Women’s Health Initiative; JHS, Jackson Heart Study; HRS, Health and Retirement Study; CI, bootstrap 95% confidence intervals

Dataset	Ancestry	*N**^a^*	Total number of SNPS*^b^*	Number of SNPs in PRS*^c^*	Partial-*R^2^* (CI, %)	European Ancestry (%)
UKB European subset (UKB_eur)	European	9,998^d^	685,475	6,052	22.4 (20.8-24)	100
HRS European (HRS_eur)	European American	10,159 (10,123)	1,515,431 (10,118,786)	7,117 (9,724)	15.6 (14.4-16.9)	100
UKB admixed African (UKB_afr)	African + European	8,700 (8,696)	685,475 (13,279,553)	6,049 (10,577)	4.1 (3.2-4.9)	13.1
WHI (WHI_afr)	African American	6,863	741,983	5,744	3.6 (2.8-4.5)	22.7
JHS (JHS_afr)	African American	1,773	702,685	5,676	3.8 (2.2-5.7)	17.7
HRS admixed African HRS_afr	African American	2,251 (2,241)	1,511,742 (10,118,786)	7,101 (9,724)	3.1 (1.9-4.6)	17.5

a number of individuals;

b number of SNPs in the intersection between genotyped (or imputed) SNPs and SNPs from the height GWAS that passed our filters;

c SNPs clumped in 100 Kb windows and with *P* < 0.0005;

a, b,c number of individuals and SNPs in imputed set in parentheses; d, randomly selected from the entire European component of the cohort.

**WHI**: This dataset contains African American and Hispanic participants. We ran unsupervised ADMIXTURE (Alexander *et al.* 2009) with k = 3 and identified 7,285 individuals with self-reported “African American” ancestry with at most 0.8 of the first ADMIXTURE component (interpreted as reflecting European ancestry), and at most 0.05 of the second (reflecting Native American ancestry; Figure S2**)**. We further filtered this set to contain individuals with at least 5% genome-wide African ancestry, phenotype availability, and height between ± 2 SD (sd) from the mean (Figure S3), resulting in 6,863 individuals (Table 1). We refer to them as “WHI_afr”. The final filter was because the public release of the WHI dataset truncates the extreme 1% of the phenotype distribution (approximately ± 2 SD) to reduce the chance of individual identifiability.

**HRS**: This dataset contains European American, African American, and Hispanic participants. We ran unsupervised ADMIXTURE with K = 3, and identified 2,322 individuals with self-reported “Black/African American” ancestry with at least 0.05 of the first ADMIXTURE component (interpreted as reflecting African ancestry), and at most 0.05 of the second ADMIXTURE component (interpreted as reflecting Native American ancestry, see the boxed area in Figure S4**).** We further filtered this set to contain individuals with at least 5% genome-wide African ancestry and sex-corrected height not less than 2 sd below the mean (Figure S3, to remove individuals with anomalously low height values), resulting in 2,270 individuals (referred to as “HRS_afr”). We also identified 10,486 individuals who self-reported “White/Caucasian” ancestry and had at most 0.05 of each of the first two ADMIXTURE components, of which 10,159 had sex-specific height above the -2 sd cutoff (“HRS_eur”, Figure S5, Table 1).

**JHS**: This dataset contains only African American participants, so we did not filter the data based on PCA or ADMIXTURE. After ancestry inference, we retained all 1,773 individuals with at least 5% African ancestry (“JHS_afr”).

GWAS results: We obtained UK Biobank summary statistics for height from the Neale Lab GWAS on 360,388 individuals of European ancestry (round 2; https://www.nealelab.is/uk-biobank, accessed April 2, 2019). We used a set of 13,586,591 autosomal SNPs that passed QC filters of INFO score > 0.8 and MAF > 0.0001. For some analyses, we used between-sibling effect sizes estimated at a subset of 1,284,881 SNPs (Cox *et al.* 2019). Table 1 shows the number of individuals and SNPs per dataset.

### Ancestry inference

For the admixed cohorts, we estimated local and genome-wide ancestry. We merged each dataset with CEU (Utah residents with Northern and Western European ancestry) and YRI (Yoruba from Ibadan, Nigeria) individuals from The 1000 Genomes Project (2015) and phased each dataset separately using HAPI-UR (Williams *et al.* 2012) with a window size of 91. We then used RFMix (Maples *et al.* 2013) to infer local ancestry, using the CEU and YRI individuals as references for European and African ancestry, respectively. We used the most likely ancestry path inferred by the Viterbi algorithm of RFMix to estimate proportions and checked that they were consistent with those obtained from unsupervised ADMIXTURE with K = 2 **(**Figure S5**)**.

### Clumping and thresholding (c+t) SNPs

We first intersected the ∼13.5 million SNPs from the UK Biobank summary statistics and the genotyped SNPs in each dataset **(**Table 1**)**. Next, we defined sets of SNPs based on a variety of clumping strategies. We clumped SNPs in physical and genetic windows using a range of p-value thresholds. Physical window sizes (in Kb) were: 1,000, 500, 100, 75, 50, 25, 10, 5. Genetic window sizes (in cM) were: 1, 0.5, 0.3, 0.25, 0.2, 0.15, 0.1. We considered SNPs below the p-value thresholds: 5×10^−7^, 5×10^−6^; 5×10^−5^ 5×10^−4^, 5×10^−3^. For each set of parameters, we followed these steps: 1) retain only SNPs below the p-value threshold, 2) select the lowest p-value SNP, 3) remove SNPs within the window around that SNP, 4) repeat steps 2 and 3 until there are no SNPs left. We also used a strategy of clumping based on empirical LD structure. We used PLINK2 (Chang *et al.* 2015) to estimate *r*^2^ between pairs of SNPs using UKB_eur (–clump-p1 0.01 –clump-r2 0.5–clump-kb 250|100| 50). Finally, we applied a strategy where we clumped SNPs in approximately independent LD blocks (Berisa and Pickrell 2016) (defined in either African or European populations). In total, we evaluated 80 pruning strategies **(**Table S1**)**.

We also calculated PRS using LDpred (Vilhjálmsson *et al.* 2015). We used the UKB_eur imputed genotypes as an LD reference panel and the UKB GWAS summary statistics for height. We estimated weights separately for the SNPs present in each dataset using both the Gibbs sampler and the infinitesimal model and evaluated the partial-*R*^2^ as described above.

For the unweighted PRS, we tested prediction for the same 80 sets of SNPs **(**Table S1**)**. We repeated these steps for analyses using effect sizes re-estimated from sibling pairs and imputed genotypes, except restricting the initial set of SNPs (before pruning/clumping) to those present in the sibling or imputed dataset. For imputed genotypes, we performed the 40 c+t strategies using the physical windows described above **(**Table S1**)**.

### Effect size estimates for African ancestry

We ran a GWAS using PLINK (Chang *et al.* 2015) on the Admixed African individuals from the UK Biobank, including sex, age, age^2^, and the first 10 principal components, computed using smartpca (Patterson *et al.* 2006), as co-variates. We then computed a chi-squared statistic for the difference between the Admixed African effect size ($\beta_{\;afr}$, with standard error $\sigma_{afr}$) we obtained and the European effect sizes from the UK Biobank ($\beta_{eur}$with standard error $\sigma_{eur}$):

$$\chi_{diff}^{2}={\left[\frac{\beta_{eur}-\beta_{afr}}{\sqrt{\sigma_{eur}^{2}+\;\sigma_{afr}^{2}}}\right]}^{2}\mathrm{(Equation 1)}$$

### PRS and odds-ratio calculation

We calculated PRS for each individual, *j*, as the weighted sum of effect sizes:

$$PRS_{j}=\overset{M}{\underset{i=1}{\sum }}\beta_{i}G_{ij}\mathrm{(Equation 2)}$$

where the sum is over all *M* SNPs used in the PRS, *G_ij_* is the effect allele dosage (0, 1 or 2) of individual *j* at SNP *i*, and $\beta_{i}$ is the estimated effect size of the effect allele at SNP *i*. To calculate unweighted PRS, we set $\beta_{i}$ to ±1 depending on whether the original $\beta_{i}$ is positive or negative.

To evaluate predictive power, we fitted a linear model of height as a function of sex, age, age^2^, genome-wide European ancestry proportion ($p_{eur}$), and PRS ($height∼sex+age+age^{2}+p_{eur}+PRS$), and compared it to a model without PRS ($height∼sex+age+age^{2}+p_{eur}$). The partial-*R^2^* between the two models gives the proportion of the phenotypic variation explained by the PRS, to which we refer as partial-*R*^2^ or predictive power, throughout. To evaluate the effect of ancestry on predictive power, we stratified each dataset into 2-4 equally sized bins (2 for JHS_afr and HRS_afr, 4 for WHI_afr and UKB_afr) based on $p_{eur}$. Next, we calculated the partial-*R*^2^ for each bin. To infer confidence intervals, we used the R package “boot” (Davidson and Hinkley 1997) to perform a percentile bootstrap over samples with 1,000 replicates. For HRS_eur we used the entire set of 10,159 individuals and calculated confidence intervals for that set. Finally, we performed a weighted regression – using the inverse of the bootstrap standard deviation as weights — of the partial-*R*^2^ values on the median proportion of European ancestry in each bin. We repeated this analysis with imputed genotypes, unweighted PRS and sibling-estimated effect sizes.

We also constructed linear combinations of PRS (Márquez-Luna *et al.* 2017). Using Equation 2, we calculated $PRS_{eur}$ using effect sizes from the UK Biobank, and $PRS_{afr}$ using the same SNPs as $PRS_{eur}$, but with effect sizes we re-estimated from the UKB_afr dataset (see above). In $PRS_{C}^{1}$, we weight $PRS_{afr}$ in all individuals by a common factor α ranging from 0-1, and in $PRS_{C}^{2}$, in addition to α, each individual’s $PRS_{afr}$ is weighted by $p_{eur}$, the proportion of European ancestry for the individual. So, for individual *j*:

$$PRS_{C}^{1}=\alpha PRS_{afr,j}+(1-\alpha )PRS_{eur,j},\text{(Equation 3)}$$

$$\;PRS_{C}^{2}=\alpha \left(1-p_{eur,j}\right)PRS_{afr,j}+(1-\alpha +\alpha p_{eur,j})PRS_{eur,j}\text{(Equation 4)}$$

We evaluated the predictive power of $PRS_{C}^{1}$ and $PRS_{C}^{2}$ in WHI_afr, JHS_afr and HRS_afr. We also used Equation 2 to calculate PRS based only on European ancestry segments of the genome (from the local ancestry analysis) and repeated the analysis of partial-*R^2^* as a function of $p_{eur}$.

Finally, we constructed a combined PRS where Admixed African effect sizes are used for SNPs falling in African ancestry regions of the genome, and European effect sizes are used for SNPs falling in European ancestry regions. For African ancestry segments, effect sizes from admixed Africans are weighted by a constant, α. So, for each haplotype in each individual, we have:

$$PRS_{C}^{3}=\text{α}\left[\underset{i\in AFR}{\sum }\beta_{i,afr}G_{i}\right]+\left(1-\alpha \right)\left[\underset{i\in AFR}{\sum }\beta_{i,eur}G_{i}\right]+[\underset{\;i\in EUR}{\sum }\beta_{i,eur}G_{i}]\text{(Equation 5)}$$,

where *G_i_* is the genotype of the *i*-th SNP, and *EUR* and *AFR* are the sets of European and African ancestry regions of the genome (specific to each individual). We then sum $PRS_{C}^{3}$ for both haplotypes of each individual, and refer to this sum as $PRS_{C}^{3}$ for simplicity.

We estimated the odds ratio for being in the upper *q*-quantile of height conditional on being in the upper *q*-quantile of PRS as:

$$OR=\;\frac{P(height_{q}|PRS_{q})}{P(height_{q})}\text{(Equation 6)}$$

where $P(height_{q})$ is the proportion of individuals in the upper *q*-quantile of height and $P\left(height_{q}\text{|}PRS_{q}\right)$ is the proportion of individuals in the upper *q*-quantile of PRS who are also in the upper *q*-quantile of height. We used standardized residuals of height after regressing out *age*, *age^2^*, *sex* for each dataset.

### Recombination rate and LD score analyses

We used recombination maps estimated in African Americans (AA_Map) (Hinch *et al.* 2011) to estimate genetic distance in 20 Kb windows using linear interpolation between genotyped points. We stratified each dataset into four equally sized bins according to recombination rate and calculated partial-*R*^2^, and 95% confidence intervals obtained by the percentile bootstrap on 3,000 replicates over samples for each bin, as described above. We then divided the values for each bin by those obtained for the full dataset, thus obtaining a relative partial-*R*^2^. In another approach, we tested for correlation between $\chi_{diff}^{2}\text{(Equation 1)}$ and LD scores (Bulik-Sullivan *et al.* 2015). We also performed the same analysis using a recombination map derived for CEU (European) individuals from the 1000 Genomes Project (Spence and Song 2019).

### Genetic and phenotypic variance analyses

We estimated the ratio of the additive genetic variance explained by the PRS SNPs as:

$$G_{PRS}=\frac{\sum_{i=1}^{M}2f_{i,afr}(1-f_{i,\;afr})\beta_{i,eur}^{2}}{\sum_{i=1}^{M}2f_{i,eur}(1-f_{i,\;eur})\beta_{i,eur}^{2}}$$ (Equation 7)

where *f_i,eur_*, *f_i,afr_*, *β_i,eur_* and *β_i,afr_* are the allele frequencies and effect sizes for each of the *M* PRS SNPs in the European or admixed African ancestry cohorts, respectively. For HRS_afr, HRS_eur, UKB_afr, and UKB_eur, allele frequencies were obtained directly from the datasets. For WHI_afr and JHS_afr, the denominator was estimated from frequencies of non-Finnish European individuals from gnomAD (Lek *et al.* 2016).

### Modeling height variance as a function of ancestry

We combined all 29,746 individuals **(**Table 1, UKB_eur excluded**)** and computed the residuals *y_i_* of the regression of height on *sex*, *dataset*, *age*, *age^2^*, sex*dataset, sex*age, sex*age^2^, dataset*age, dataset*age^2^. We then fitted a linear model for residual height as a function of the ancestry of individual *j* ($p_{\;j,\;eur}$) and allowed the variance to vary linearly with ancestry:

$$y_{j}=\mu +\beta p_{j,\;eur}+\varepsilon_{j};\;\;\;\varepsilon_{j}∼N(0,\sigma^{2}+\gamma p_{j,\;eur})$$ (Equation 8)

for model coefficients $\mu ,\beta ,\sigma^{2}$ and γ. We fit this model using the GAMLSS package (Rigby and Stasinopoulos 2005) in R (R Core Team 2017).

### Local differences in allele frequency

We calculated allele frequencies for all variants in the HRS_afr and HRS_eur subsets separately. We defined 10 Kb windows around each PRS SNP and calculated the mean squared frequency difference between subsets for all the SNPs contained in the window. We explore the effect size difference for AFR and EUR (Equation 1) for each PRS SNP as a function of the mean squared frequency difference in the window surrounding each SNP. We then repeated the analysis for 6kb windows.

### Data availability

Scripts developed specifically to perform the data analyses reported in this work are available at: https://github.com/mathilab/PRS_Height_Admixed_Populations. Genotype and phenotype data were obtained from UK Biobank or dbGaP. File S1 contains 14 supplementary figures. Table S1 contains 20 sheets. Sheets 1-6: Different SNP sets generated by clumping and their PRS values. Effect sizes from 360K European ancestry individuals from the UK Biobank. Data from genotype arrays. Sheets 6-12: Different SNP sets generated by clumping and their PRS values. Effect sizes estimated from ‘White British’ sibling pairs from the UK Biobank. Data from genotype arrays. Sheet 13: Different SNP sets generated by clumping and their PRS values. Data from imputed datasets. Sheets 14-19: Different SNP sets generated by clumping and their unweighted PRS values. Sheet 20: Difference between PRS values for 1000G super-populations (Africa, Europe) for different sets of SNPs. Supplemental material available at figshare: https://doi.org/10.25387/g3.12795887.

## Results

### Constructing polygenic risk scores

We tested 81 approaches to PRS construction, including five different p-value cutoffs and 15 window sizes, pairwise *r*^2^ and LD blocks inferred for African and European populations, and the infinitesimal model of LDpred. Among the clumping and thresholding (c+t) strategies, increasing the p-value cutoff and window sizes improves prediction **(**Figure S6 and Table S1**)**. LD clumping yields higher predictive power but depends on prior knowledge of the population-specific LD structure and has the highest difference in PRS between 1000 Genomes European and African Populations **(**Table S1**)**. Approximately independent LD blocks (Berisa and Pickrell 2016) yields small sets of SNPs that explain almost as much variation as the larger (23-37 times) LD clumping sets, but also rely on knowledge of the LD structure, as does LDpred. Thus, we focused on strategies that are independent of LD and chose a set of SNPs using a p-value threshold of 0.0005 and a physical window of 100 Kb, which includes ∼5,600-7,100 SNPs (Table 1) and obtains partial-*R^2^* values close to the LD clumping strategies while requiring about 10-fold fewer SNPs. In any case, results for other strategies are qualitatively similar **(**Table S1, Figure S6, Figure S7**)**.

### Predictive power increases linearly with proportion of European ancestry

We estimated the predictive power of the PRS in each dataset. Partial-*R^2^* was 22.4% in UKB_eur, and 15.6% in HRS_eur (Table 1). Because the 9,998 UKB_eur individuals analyzed here were also in the discovery GWAS, we use the HRS_eur dataset throughout the paper as representative of European ancestry. In the admixed cohorts (WHI_afr, JHS_afr, HRS_afr, UKB_afr), partial-*R^2^* was 3.1–4.1%, or between 3.8- to 5-fold lower than in HRS_eur, consistent with previous observations (Ware *et al.* 2017; Martin *et al.* 2019).

**Figure 1:**
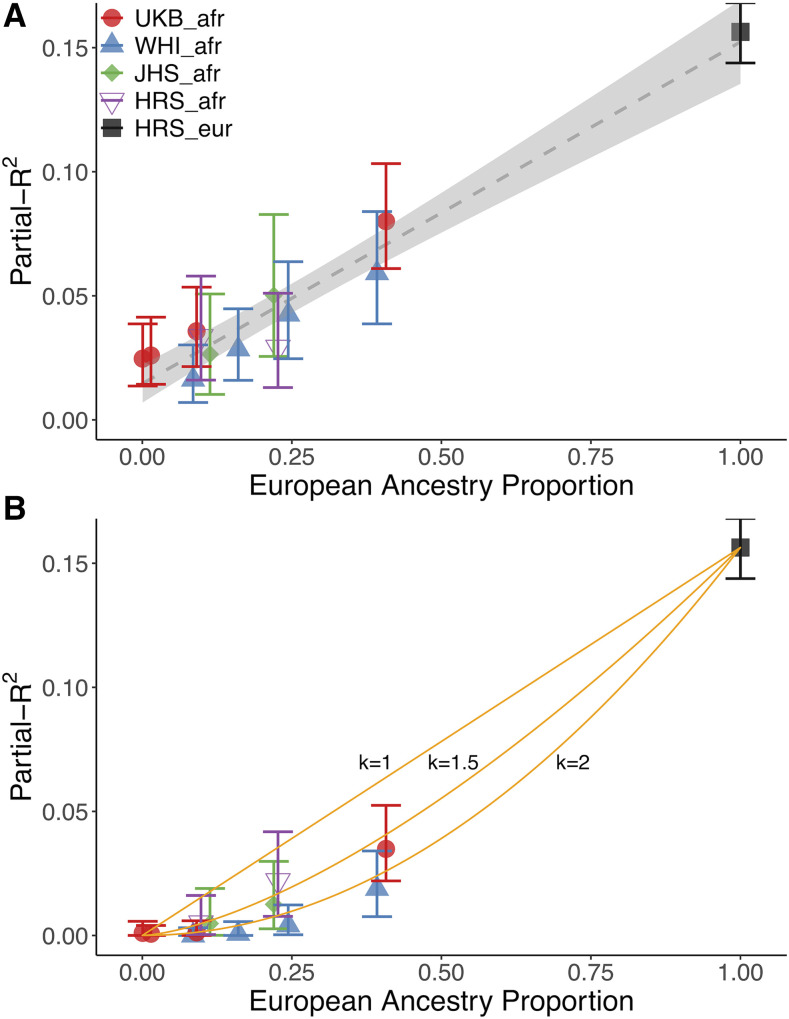
Partial-*R*^2^ as a function of European ancestry in admixed populations. Each admixed dataset is split up into quantiles of European ancestry proportion. Each quantile has between 886 and 2,175 individuals, and plotted values represent the median of each bin. Vertical bars represent 95% confidence intervals estimated from case resampling bootstrap (1,000 replicates). The dashed line shows the regression with standard errors shaded in light gray. A: Using all segments of the genome. B: Using only European ancestry segments. The orange lines represent the equation $\text{y}=0.15{\text{p}}_{\text{eur}}^{\text{k}}$, for k={1,1.5,2}. k = 1 and k = 2 represent the extreme cases where the predictive power in admixed individuals comes entirely from European ancestry segments of the genomes (k = 1) or is uniformly distributed across the whole genome (k = 2).

Stratifying individuals in each cohort by their average genome-wide ancestry, we find that partial-*R*^2^ increases linearly with European ancestry (by 1.3% for each 10% increase in European ancestry; Figure 1A). We estimated the partial-*R*^2^ in individuals with no European ancestry (*i.e.*, the intercept of this regression) to be 1.5% (S.E.=0.3%). This result is robust to the set of SNPs used in the PRS, with intercepts ranging between -1% and 2.5%, depending on the pruning strategy **(**Figure S6**)**. We observe the same pattern when using the LDpred infinitesimal model **(**Figure S7**)**. Relevant for clinical interpretation, the odds-ratio for ‘tallness’ in the tails of the PRS distribution is also lower in the admixed populations than in the European ancestry population, although only 2.3-fold on average across populations between the highest and lowest 5% of the European ancestry spectrum (95^th^ quantile of PRS distribution), compared to the 3.8- to 5-fold difference in partial-*R^2^* **(**Figures S8, S9**)**.

We next restricted the PRS SNPs to those found in segments of the genome inferred to have European ancestry **(**Figure 1B**)**. If the predictive power of the PRS came entirely from these segments, then we would expect the relationship between ancestry and partial-*R*^2^ to be the same as when we used the whole genome (*i.e.*, linear as in Figure 1A). On the other hand, if the predictive power were uniformly distributed across the genome, we would expect a quadratic relationship: the partial-*R*^2^ of the whole genome (which scales linearly with ancestry) would be multiplied by the proportion of the genome in European ancestry segments (*i.e.*, ancestry). Our observations are intermediate to these extremes (Figure 1B). We conclude that the predictive power of the PRS is enriched in, but does not entirely come from, the European ancestry segments of the admixed genomes, suggesting that the ancestry-specific interactions might play a role.

**Figure 2:**
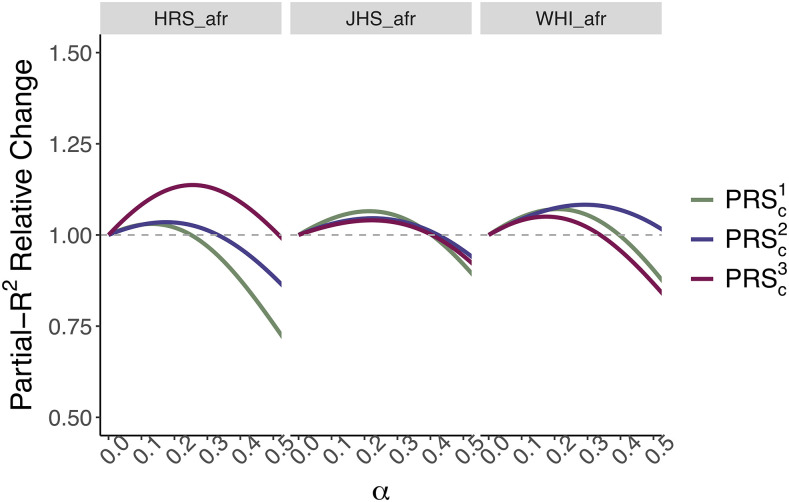
Predictive power of linear combinations of PRS. Relative partial-*R*^2^ increase for HRS_afr (N = 2,251), JHS_afr (N = 1,773), and WHI_afr (N = 6,863) from three linear combinations of *PRS*_eur_ and *PRS*_afr_. The dashed line represents no difference in performance between the linear combinations and *PRS*_eur_. For $PRS_{\text{c}}^{1}$ and $PRS_{\text{c}}^{2}$, α represents the constant weight given to the African component across individuals. $PRS_{\text{c}}^{2},$ in addition to α, weights the African component based on individual African ancestry. $PRS_{\text{c}}^{3}$uses European effect sizes for PRS effect alleles falling in European ancestry segments, and a linear combination of European and African effect sizes (weighted by α) for PRS effect alleles falling in African ancestry segments (Equations 3-5).

Next, we explored whether combining ancestry-specific PRS could improve predictive power, as suggested by Márquez-Luna *et al.* (2017). A simple linear combination of PRS improves partial-*R*^2^ in WHI_afr (3.6–3.9%), JHS_afr (3.8–4.1%), and HRS_afr (3.1–3.2%) **(**Figure 2). Weighting the combination by the ancestry proportion of each individual produces a similar improvement: 3.9% for WHI_afr, 4% for JHS_afr, and 3.2% for HRS_afr (Figure 2). Finally, we used local ancestry information to construct a PRS using ancestry-specific effect sizes at each SNP (Figure 2). This approach produces a similar improvement to the global ancestry weighted PRS, with a partial-*R*^2^ increase between 0.1 and 0.3% across datasets. While these absolute improvements are modest, this is likely due to GWAS sample size discrepancy (*N* = 8,700 Admixed African and *N* = 361,194 European). With larger African ancestry GWAS, we expect that we would be able to improve the PRS performance in the admixed populations with this approach.

### Why does predictive power vary with ancestry?

Several explanations have been proposed to explain why the predictive power of PRS is lower in non-European ancestry populations. These include differences in LD patterns, the allele frequency of PRS variants, additive genetic variance, gene-gene (G×G) and gene-environment (G×E) interactions in different populations, and biases in the discovery cohort. In this section, we evaluate the impact of some of these factors on PRS-based phenotypic prediction.

### Differences in the site frequency spectrum

Differences in the frequencies of the tag variants could lead to different partial-*R*^2^ values across ancestries. Because GWAS have more power to detect more common variants, one hypothesis is that the PRS will tend to contain variants that are more common in European than African ancestry backgrounds–resulting in systematically lower predictive power in African ancestry populations. We tested this by comparing the ratio of the additive genetic variance contributed by the variants used in the PRS in the European and the admixed datasets (Equation 7). This ratio is the relative difference we would expect if the effect sizes and LD structure were the same across ancestries, and only allelic frequencies differed. We estimate this ratio to be: 0.78 (UKB), 0.92 (HRS), 1.04 (JHS), and 1.07 (WHI), suggesting that at most 8% of the decrease in partial-*R^2^* (in non-UKB samples) can be explained by differences in the site frequency spectrum (SFS). JHS and WHI have fewer SNPs genotyped than HRS and UKB (Table 1). One possibility is that those arrays are more biased toward SNPs that are common across ancestries.

### Differences in the total genetic variance

A related possibility is that SFS differences might affect not just the variance explained by the PRS SNPs, but also the total genetic variance. Genome-wide heterozygosity in European ancestry populations is approximately 30% lower than in West African ancestry populations (The 1000 Genomes Project Consortium 2015). If this were true for SNPs that causally affect height, then the additive genetic variance of those SNPs would also be 30% lower. Assuming constant environmental variance and height heritability to be 80%, it would follow that that the European phenotypic variance would be about 24% lower (0.8*0.7+0.2). Furthermore, the phenotypic variance in admixed populations would vary linearly with ancestry. In this case, the PRS could capture the same absolute amount of phenotypic variance, but the proportion of variance explained would be higher in European ancestry populations. However, we find that phenotypic variance does not vary significantly with genome-wide ancestry proportion once we regress out sex, age, age^2^, dataset, and all their interactions (*P* = 0.133, Figure S10).

### Population-specific linkage disequilibrium (LD) patterns

Variants identified by GWAS are not generally themselves causal but rather are linked to the causal variant(s). Linkage disequilibrium patterns are known to differ between populations, suggesting that tag SNPs discovered in the original European ancestry GWAS may be less efficient at tagging the causal variants on non-European ancestry haplotypes. Using GWAS variants detected in an exclusively European ancestry cohort would thus result in a reduced partial-*R*^2^ in admixed African populations when compared to European ancestry populations.

**Figure 3:**
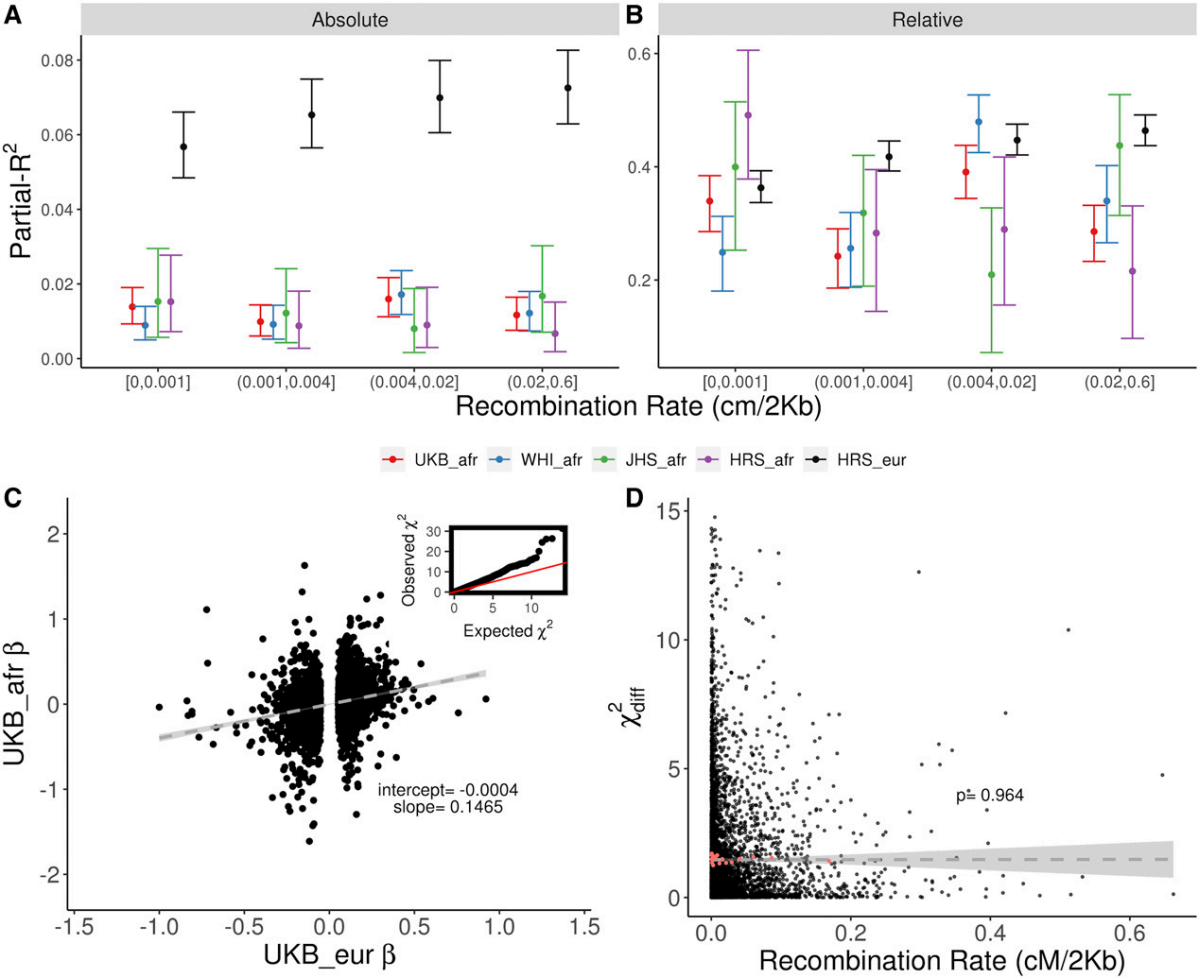
Effect of recombination rates on predictive power. A and B: PRS SNPs from each dataset were binned into quartiles of African American recombination rate. Absolute (A) and relative (B) partial-*R*^2^ for subsets of SNPs divided by the total partial-*R*^2^ for each dataset (Table 1). Vertical bars show 95% bootstrap confidence intervals. C: correlation between PRS SNPs effect sizes from Europeans and Admixed Africans in the WHI_afr dataset. The inset shows a qq-plot of ${\text{χ}}_{\text{diff}}^{2}$ for PRS SNPs. The dashed line shows the regression with standard errors shaded in light gray. D: X-axis, recombination rate in cM/20Kb. Y-axis, statistic for the difference in betas between European and African ancestries (Equation 1) in WHI_afr. Cut-off at 15 for display purposes excludes 10 data points. The dashed line shows regression with standard errors shaded in light gray. Red points represent the median recombination rate for each of 20 quantiles of recombination rate.

If LD differences between African and European haplotypes drive the pattern seen in Figure 1, then a PRS constructed from SNPs in low recombination regions should be more transferable than a PRS constructed from SNPs in high recombination regions of the genome. When we bin PRS SNPs into quartiles of recombination distance and calculate PRS for SNPs in each bin, we see that partial-*R*^2^ for the admixed cohorts tends to decrease between the first and fourth quantiles of recombination **(**Figure 3B**)**, suggesting that differences in LD do play a role in reducing prediction in non-Europeans. On the other hand, we note that, even for the quartile of lowest recombination, the reduction in partial-*R*^2^ for admixed individuals is substantial – 76% on average across datasets – compared to 84% for the fourth quantile **(**Figure 3A**)**. Thus, even if all PRS variants were from low recombination regions, we would still observe a substantial reduction in predictive power. We performed the same analysis using a recombination map derived from the 1000 Genomes CEU population (Spence and Song 2019) and found consistent results **(**Figure S11). One potential confounding factor in this analysis is that causal variants in low recombination regions might be better tagged than those in high recombination regions, which would reduce the relative performance in high recombination regions. However, since we find little evidence of difference, we conclude that this is unlikely to be a major factor.

A second prediction is that the difference between effect sizes estimated in European and African ancestry populations should be larger in regions of high recombination. To test this, we evaluated whether effect sizes estimated directly from admixed individuals differ from the original (European) effect sizes (Figure 3C) and whether these differences are correlated with recombination in the regions in which they are located. We find no significant correlation (ρ=0.0005, *P* = 0.97) between $\chi_{diff}^{2}\;$and local recombination rate (Figure 3D), and a small positive correlation between $\chi_{diff}^{2}\;$and European LD scores (Bulik-Sullivan *et al.* 2015) (ρ = 0.0292, *P* = 0.0379) (Figure S12).

**Figure 4:**
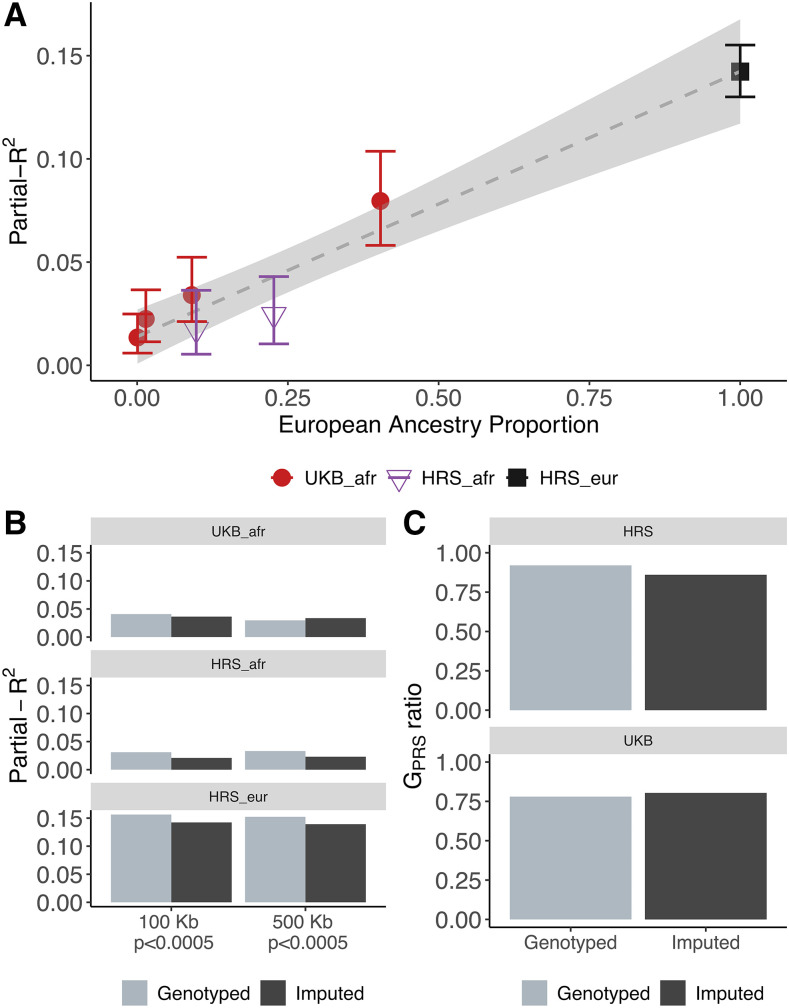
Imputed data. A: Partial-*R*^2^ as a function of European ancestry, where each admixed dataset is split up into quantiles of European ancestry proportion. Vertical bars show 95% bootstrap confidence intervals estimated from case resampling bootstrap (1,000 replicates). The dashed line shows the regression with standard errors shaded in light gray. B: Partial-*R*^2^ for two clumping strategies (100 and 500Kb windows with either *P* < 0.005 or *P* < 0.00005) for imputed and genotyped sets of SNPs. C: additive genetic variance ratio for PRS SNPs (Equation 7).

A third prediction is that, if differences in partial-*R*^2^ are driven by differences in ability to tag the causal variant, then PRS constructed from imputed genotypes should see a smaller decrease in predictive power than those constructed from genotype array data. Using imputed genotypes for the HRS and UKB cohorts, we find that the relationship between ancestry and partial-*R*^2^ is the same for imputed and array data suggesting that this is not the case (Figure 4A). In fact, the absolute performance of imputed and genotyped data are similar (Figure 4B), consistent with previous observations (Ware *et al.* 2017). Moreover, the ratio of genetic variance explained by PRS SNPs is similar for imputed and genotyped data (Figure 4C). These results suggest that the genotyping arrays are efficient at capturing the SNP heritability, at least for the c+t PRS strategies that we used. It is important to note that different datasets use different arrays, and a different pattern could be observed for other datasets. We conclude that, while differences in LD do affect PRS transferability, they are not the only factor affecting the relationship between ancestry and predictive power.

### Differences in marginal effect size

**Figure 5:**
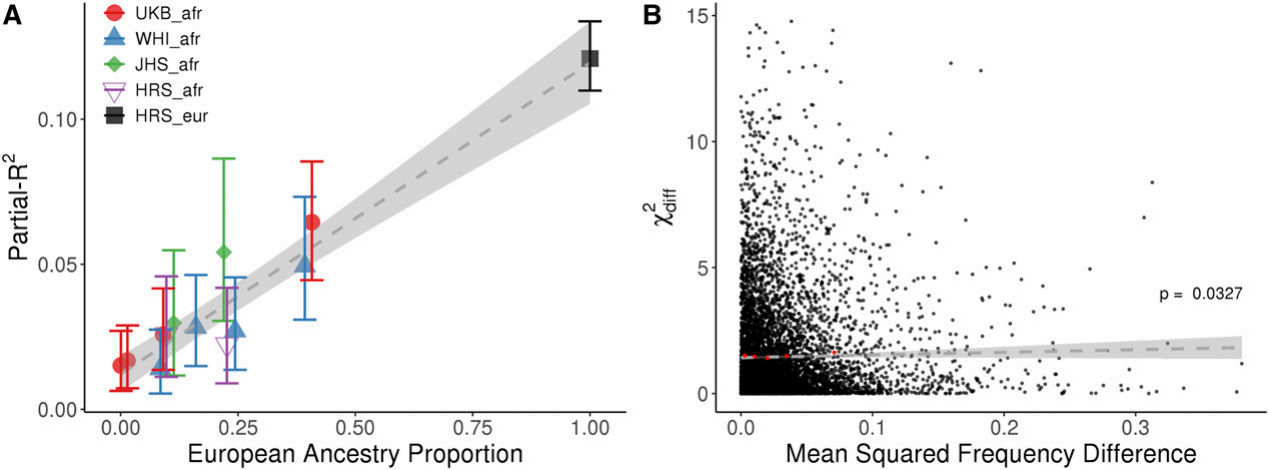
Unweighted PRS and the effect of local allele frequency differences on effect size differences. A: Partial-*R*^2^ for an unweighted PRS that uses the sign but not the magnitude of each SNP effect (Methods). Each admixed population is split up into quantiles of European ancestry proportion. Vertical bars represent 95% confidence intervals estimated from a case resampling bootstrap (1,000 replicates). The dashed line shows the regression with standard errors shaded in light gray. B: X-axis, mean squared frequency difference for PRS SNPs for European and African ancestries in a 6 Kb window around each PRS SNP (Methods). Frequencies were calculated per dataset (HRS_eur, HRS_afr) for the causal allele. Y-axis, statistic for the difference in betas between European and Admixed African ancestries (Equation 1) in WHI_afr. Cut-off at 15 for display purposes excludes 15 data points. Dashed line shows the regression with standard errors shaded in light gray. Red points represent the median recombination rate for each of 5 quantiles of mean squared difference.

The marginal effect size at a PRS SNP depends on the cumulative effects of the causal variants that it tags. Therefore, marginal effect sizes at PRS variants across ancestries might differ for several reasons, including local epistasis or allelic heterogeneity. When we ignore effect sizes and calculate the unweighted PRS, we see a similar pattern to Figure 1A (Figure 5A), suggesting that not only marginal effect sizes but even directions differ between ancestries. That we can improve the predictive power of PRS by including effect sizes re-estimated in African ancestry populations (Figure 2) also indirectly supports the role of effect size differences. Finally, we find that allele frequencies differ more between African and European populations around SNPs with larger effect size differences, although the effect is rather small (ρ = 0.0005; *P* = 0.0327; Figure 5B, Figure S13). These results suggest that marginal effect sizes differ across ancestries and that this is one of the factors underlying the reduction in predictive power.

### Residual population stratification in the discovery cohort

Despite statistical methods to control for population stratification, it continues to be a confounding factor in the analysis of GWAS results (Berg *et al.* 2019; Sohail *et al.* 2019) and could inflate predictive power in European relative to non-European ancestry cohorts. To test this, we used effect sizes at PRS SNPs re-estimated within sibling pairs from the UK Biobank (Cox *et al.* 2019). This approach should remove much of the effect of population stratification. We find that the linear relationship between ancestry and predictive power is similar to that observed for the GWAS PRS (Figure S14), albeit absolute partial-*R*^2^ values are lower across all datasets (Figure S14, Table S1**)**. We conclude that residual population structure in the UK Biobank GWAS results does not drive differences in predictive power across ancestries.

## Discussion

Polygenic scores may become a useful tool in translational and precision medicine, but are limited by their lack of applicability in non-European ancestry populations (Ware *et al.* 2017; Martin *et al.* 2019; Marnetto *et al.* 2020). Consequently, much of the potential of genomic disease risk profiling is restricted to European ancestry populations. Here, we show that the predictive power of PRS is approximately proportional to ancestry in populations of admixed European and African ancestry. We focused on the clumping and thresholding approach to PRS construction, although we saw consistent results with LDpred’s infinitesimal model **(**Figure S7). More sophisticated approaches provide limited improvement in predictive power, require additional assumptions about LD structure or other parameters, and do not necessarily lead to substantial improvements in predictive power or transferability (Kulm *et al.* 2020).

We show that differences in LD structure and SFS affect the transferability of PRS but do not explain the full magnitude of the decrease. Our results are broadly consistent with simulation studies showing that these two factors are expected to decrease variance explained when the test cohort has different ancestry from the GWAS cohort and, specifically, that together they explain up to 72% of the loss of accuracy in prediction between European and African ancestry (Wang *et al.* 2020). Moreover, our findings agree with estimates that the *trans*-ancestry correlation in effect sizes for height is less than 1 (between 48 and 71%) (Veturi *et al.* 2019), and therefore that the marginal effect sizes at PRS SNPs are systematically different across ancestries. We interpret this as evidence that *cis*-epistasis or allelic heterogeneity – which mimics epistasis (Wood *et al.* 2014) – contribute to these differences. However, this may not, in general, be the only contributing factor. Gene-by-environment (Veturi *et al.* 2019; Mostafavi *et al.* 2020) and gene-by-ancestry interactions may also contribute, and the relative importance of these mechanisms remains to be quantified.

By incorporating effect sizes from admixed populations in a linear combination of PRS, we are able to improve predictive power, in agreement with previous findings (Márquez-Luna *et al.* 2017; Marnetto *et al.* 2020). Although the inclusion of individual and local ancestry information yielded only a modest increase in predictive power, this is likely due to the low sample size of our African-ancestry GWAS. With better-powered GWAS to estimate ancestry-specific effect sizes, the improvement should be more extensive. In agreement with this, a recent study showed relatively higher improvement in height prediction when using ancestry specific effect sizes from a moderately large GWAS (N = 160,000) for an East Asian ancestry population (Marnetto *et al.* 2020). This suggests that large cohorts of diverse ancestries and admixed populations are needed to make PRS broadly applicable.

Our approach has several limitations. In order to disentangle the factors affecting PRS generalizability across ancestries, we focused on height – a model trait due to its high polygenicity and heritability – in recently admixed cohorts of highly diverged European and African ancestry in the US and the UK. We expect that our insights will transfer to some extent to other traits, ancestries, and cohorts, but there may be significant differences. For example, genetic architecture, local adaptation, and environmental factors, to name a few, might differ, and our results might not directly apply to the same extent. A related issue is that there is variation across the African ancestry samples used in this study, although we treated African ancestry as derived from a single population. On the other hand, most of the ancestry of the populations that our cohorts are drawn from is West African, even other African ancestries are largely symmetrically related to Europe, and in these cohorts, the admixture proportion is the major component of variation (Zakharia *et al.* 2009; Tishkoff *et al.* 2009; Patin *et al.* 2017). Ideally, we would have enough individuals and reference panels to properly integrate the different components of African ancestry into our analyses, and this is an essential problem for future research.

Another limitation of our approach is the difficulty of distinguishing the effects of correlated variables. For example, the site frequency spectrum, the recombination rate, the density and effect sizes of GWAS hits, and the effectiveness of tag SNPs are all correlated. While we can separate these effects in some cases, our results are likely still confounded to some extent. In simulations, we can control and quantify these effects. However, this requires realistic simulations of complex traits in admixed populations. Developing such simulations is another important future goal. A related issue is that there are both biological and environmental factors that are correlated with ancestry. Local ancestry analysis can control for many of these effects (*e.g.*, Figure 1B), but it remains a confounder for analyses based on genome-wide ancestry. Overall, our results exclude some possibilities and indicate what are likely the most relevant factors, but we are still a long way from a quantitative understanding of their relative importance.

In summary, leveraging information about each associated variant’s local ancestry background is a promising way to improve transferability, albeit that, too, requires larger non-European cohorts to estimate effect sizes. Though we focused on cohorts of recent admixed European and African ancestry, additional work is required to characterize the transferability of PRS both in populations with more complex recent admixture, as well as in populations that are more anciently admixed. While we showed that different factors each play a modest role in PRS generalizability, there is much room for advances in approaches such as ours as more diverse GWAS datasets become available.
